# Rutin and *Physalis peruviana* Extract: Population Pharmacokinetics in New Zealand Rabbits

**DOI:** 10.3390/pharmaceutics16101241

**Published:** 2024-09-24

**Authors:** Gina Paola Domínguez Moré, Diana P. Rey, Ivonne H. Valderrama, Luis F. Ospina, Diana Marcela Aragón

**Affiliations:** 1 Centro de Servicios Farmacéuticos y Monitoreo de Fármacos, Facultad de Química y Farmacia, Universidad del Atlántico, Carrera 30 # 8-49, Puerto Colombia 081001, Colombia; ginadominguez@mail.uniatlantico.edu.co; 2 Departamento de Farmacia, Universidad Nacional de Colombia, Av. Carrera 30 # 45-03 Edif. 450, Bogotá 111321, Colombia; dpreyp@unal.edu.co (D.P.R.); ihvalderramap@unal.edu.co (I.H.V.); lfospinag@unal.edu.co (L.F.O.)

**Keywords:** rutin, population pharmacokinetic, *Physalis peruviana*, PK interactions

## Abstract

**Background/Objectives:** An extract of calyces from *Physalis peruviana* with hypoglycemic activity is being considered as a potential herbal medicine. Preclinical pharmacokinetics (PK) studies of the extract in rats, focusing on plasma concentrations of its main compound, rutin, and its metabolites, revealed PK interactions in the extract matrix that improved the absorption of rutin metabolites compared to the pure compound, among other PK effects. This research aimed to study the PK of rutin alone and in the extract and assess potential PK interactions in the extract matrix on the flavonoid and its metabolites in rabbits, a nonrodent species; **Methods:** Animals received pure rutin or extract orally and intravenously. The PK analysis used noncompartmental and population pharmacokinetics (popPK) methods, and simple allometry was applied to predict human PK parameters; **Results:** The rutin concentration–time profile fit a two-compartment model with first-order elimination, while its metabolites fit a double first-order absorption model. The extract matrix led to increased absorption, distribution, and elimination of rutin as well as increased bioavailability of its metabolites in rabbits; **Conclusions:** The popPK model defined the equations for PK parameters describing these findings, and the increased volume of distribution and clearance of rutin was maintained in human predictions. These results will support the development of a new herbal medicine.

## 1. Introduction

Herbal medicine has historically been used, but at the present, it is also considered a promising future medicine, as evidenced by the growing popularity of its prescription and use worldwide [1,2]. The safety and efficacy of herbal medicines are frequently supported by their traditional use, but when the information is not available or is insufficient, the research and development process of these products is no different from that for synthetic drugs. Consequently, studies describing the pharmacodynamics (PD) and pharmacokinetics (PK) of active compounds in herbal preparations are increasingly available [3,4,5]. As with synthetic drugs, preclinical studies of new herbal medicines are recommended to include at least two species, typically one rodent and one nonrodent [6]. 

The development of new medicinal products based on plants requires consideration of PD and PK interactions, both among the known and unknown compounds present in the same ingredient, and with other herbs or drugs [7,8]. PK interactions in a single herbal drug can alter the absorption, distribution, metabolism, or excretion of its individual compounds, which is closely related to their PD. Therefore, investigating these interactions is crucial, and population pharmacokinetics (PopPK) is an approach that can facilitate their identification. Although the main applications reported for PopPK refer to conventional drugs, this approach has also been used to investigate covariates that impact the PK properties of marker compounds in herbal medicines [9,10,11,12], to explore herb–drug interactions [13,14,15,16], and, more rarely, to explore interactions within a single herbal formulation [17].

Our group has obtained an ethanolic extract of calyces from *Physalis peruviana* with hypoglycemic activity [18,19,20,21]. This plant is widely cultivated in Colombia, particularly in the departments of Cundinamarca, Boyacá, Antioquia, and Nariño, and its calyx, which naturally protects the edible fruit, often becomes postharvest waste. As part of the development process for a new medicinal product based on this extract, studying its PK is essential. According to international regulatory guidelines, the PK of herbal medicines can be studied by monitoring the plasma concentration of selected compounds within the extract, chosen as markers based on their pharmacological activity or availability as analytical standards. This approach is necessary due to the challenges in quantifying or even knowing the complete chemical composition of plant extracts [7]. Moreover, given the potential for PK interactions among components within a single herbal formulation, our group considers that a more comprehensive understanding of the PK of the extracts can be achieved by comparing the marker within the extract matrix to the marker in its pure form. 

For the extract studied in this research, the bioactive flavonoid quercetin*-3-O-*rutinoside (rutin) was fully identified as its main compound and selected as a marker for studying its PK [19,22]. Our group previously investigated the effects of the extract matrix on the PK of rutin and its glucuronide and sulfate metabolites, quercetin*-3-O-*glucuronide (Q3OG) and quercetin*-3-O-*sulfate (Q3OS), in rats. PK interactions among the components of the extracts were evidenced by differences in the plasma concentration–time profile of rutin, Q3OG, and Q3OS, along with increases in their distribution volume (Vd), clearance (*Cl*), rate constant of absorption (*ka*), and systemic exposure compared to profiles and parameters obtained for pure rutin [17]. Complementing the previous work, the current research aimed to study the PK of rutin alone and within the extract, and to assess potential PK interactions of the extract matrix on the flavonoid and its metabolites in rabbits, a nonrodent species, applying both noncompartmental analysis (NCA) and PopPK. The estimated parameters were used to predict a possible matrix effect in humans by simple allometry. 

## 2. Materials and Methods

### 2.1. Materials

Analytical standards of rutin trihydrate (89.0% of anhydrous rutin), quercetin (Quer, 95.0%), Q3OG (98.1%), and chrysin (99%) were obtained from Sigma-Aldrich (Deisenhofen, Germany). Enzymes glucuronidase/arylsulfatase and dimethyl sulfoxide (DMSO) were also sourced from Sigma-Aldrich. Water was purified using a Milli-Q system from Millipore (Bedford, MA, USA). UHPLC-grade solvents (methanol and acetonitrile) and all other reagent-grade chemicals were procured from Merck (Darmstadt, Germany).

### 2.2. Methods

#### 2.2.1. Plant Extract

The extract of calyces from *P. peruviana* was prepared following standardized protocols established by our group [20]. Calyces were collected in Granada (Cundinamarca, Colombia), and taxonomist Parra C. identified the plant material, which was archived as a voucher specimen (COL 512200) in the Herbarium of the National University of Colombia. The extract was obtained by percolation over 72 h of dried and ground calyces with 70% ethanol at a drug to solvent ratio of 1:15 (g:mL). The alcohol in the percolated product was evaporated under reduced pressure, and the concentrate was lyophilized. Finally, the content of rutin was quantified at 14.80 ± 0.3 µg/mg using the validated HPLC method described by Cardona et al. [20].

#### 2.2.2. Animals

Twenty male New Zealand White rabbits were obtained from Sentagro E.A.T (Cachipay, Cundinamarca, Colombia), who certificated their origin and traceability. The animals were between 9 and 10 weeks old, weighing between 1.8 and 2.2 kg. They were allowed to acclimate for at least 5 days under constant temperature conditions (22 ± 1 °C), with light/dark cycles of 12 h, at the animal facility of the Department of Pharmacy of the Universidad Nacional de Colombia (UNAL), where the experiments were conducted. They were provided with water and food ad libitum until 12 h prior to the experiment, when they were fasted. The study protocol was approved by the ethics committee of the science faculty of the UNAL (Act 06, 2015, project 40831, 22 June 2015).

#### 2.2.3. Study Design

Rabbits were divided into four groups to receive either pure rutin or *P. peruviana* extract intravenously (i.v.) or orally (p.o.). The dose of rutin i.v. was set at 0.37 mg/kg, based on the highest quantity of the compound able to dissolve in 1 mL of the diluent used. The dose of extract i.v. was 100 mg/kg, a reference dose for animal experimentation in our group, which also allowed the solubilization of rutin in the extract within the administration volume [23]. Oral rutin was administrated at a dose of 100 mg/kg, and oral extract was administrated at a dose of 500 mg/kg, which is an active dose from prior experiments [18,24]. Based on the rutin content in the extract, the doses of extract used were equivalent to 1.48 mg/kg and 7.4 mg/kg of rutin for the i.v. and p.o. experiments, respectively.

For intravenous administration, solutions of rutin in sodium chloride solution (0.9% NaCl) were prepared at a concentration of 0.74 mg/mL, using DMSO (<0.1% of the final volume) to dissolve the compound. Meanwhile, the extract was directly dissolved in 0.9% NaCl at a concentration of 200 mg/mL. For oral administration, pure rutin and the extract were suspended in water near the time of the experiment to achieve concentrations in suspension of 200 and 1000 mg/mL, respectively. Vortexing and sonication were applied 3–4 times for 15 min each to all preparations. Each animal received 0.5 mL/kg of the treatment via the marginal ear vein (i.v.) or by oral gavage (p.o.).

**Figure 1:**
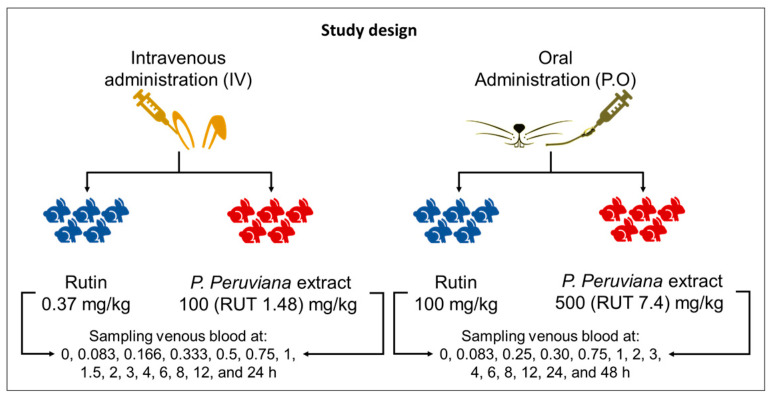
Study design. Rabbits (*n* = 5) received a dose of either pure rutin or *P. peruviana* extract, administered orally and intravenously. Blood samples were collected from the animals over a period of 24–48 h.

Samples of blood (approximately 250 µL) were withdrawn from the marginal ear vein and collected in heparinized tubes at various time points. For experiments with i.v. administration, sampling was conducted at times 0, 0.083, 0.166, 0.333, 0.5, 0.75, 1, 1.5, 2, 3, 4, 6, 8, 12, and 24 h. For experiments with p.o. administration, sampling was performed at times 0, 0.083, 0.25, 0.30, 0.75, 1, 2, 3, 4, 6, 8, 12, 24, and 48 h (Figure 1). The blood samples were then centrifuged at 3200× *g* and at 4 °C for 15 min to separate plasma, which was transferred to a new tube. The plasma was promptly acidified to pH 4 using 0.5 µL of 42.5% formic acid and then frozen at −80 °C until further analysis.

#### 2.2.4. Rutin and Rutin Metabolites Quantification

The quantification of analytes was carried out using a validated UHPLC-UV method on a Chromaster RS chromatograph (Hitachi, Tokyo, Japan). Briefly, plasma samples (100 µL) were supplemented with the internal standard, chrysin (1000 ng/mL), and divided into two tubes. One tube was immediately extracted and injected into the chromatographic system to quantify rutin. The plasma in the other tube first underwent a deconjugation reaction with 120 Fishman units of a mixture of the enzymes β-glucuronidase/arylsulfatase at pH 5.5 and 37 °C for 30 min. The product of the reaction, Quer, was also quantified, representing rutin metabolites Q3OG and Q3OS. The analytes in the plasma samples, rutin and Quer, were extracted using the protein precipitation method with twice the volume of methanol; proteins were then separated by centrifugation at 1200× *g* and 4 °C for 15 min, and the supernatant was used for instrumental analysis.

In the chromatographic system, the mobile phase consisted of a gradient from 75% to 65% of 0.1% formic acid in water complemented with 0.1% formic acid in acetonitrile. The gradient was completed in 15 min at a flow rate of 0.5 mL/min. The stationary phase was a Kinetex^®^ EVO C18 column, 100 × 2.1 mm and 2.6 µm (Phenomenex, Torrance, CA, USA), maintained at 30 °C during the runs. The UV detector was set to 260 nm, and the injection volume was 6 µL. 

This method demonstrated linearity, accuracy, precision, and stability within the concentration range of 100 to 10,000 ng/mL. Samples with concentrations above the upper limit were diluted with blank plasma before processing; accuracy and precision under such conditions were also previously confirmed. Information about the parameters of method validation, calibration curves, and chromatograms of reference is available in our previous publication [17]. Selectivity, accuracy, and precision from rabbit plasma were confirmed for the runs in this study (Figure S1 and Table S1).

### 2.3. Data Analysis

#### 2.3.1. Noncompartmental Analysis

Concentrations–time data were tabulated and processed using MonolixSuite™ 2024R1 (Lixoft^®^, Paris, France). NCA was performed using the PKanalix^®^ module.

Peak concentration (*Cmax*) and time for peak concentration (*Tmax*) were directly obtained from the data of each individual. Other PK parameters in NCA were determined using the linear trapezoidal integral mode of the software. The parameters of interest included the volume of distribution at the steady state (*Vss*) and at the terminal phase (*Vz*); *Cl*, area under the curve extrapolated to infinite (*AUC*_0–∞_), first-order rate constant associated with the terminal portion of the curve (*λz*), terminal half-life (*t*_1/2_), and mean residence time (*MRT*). 

*AUC*_0–∞_ values were used to determine the metabolized fraction (*Fmet*), the oral bioavailability of rutin (*F*), and the relative bioavailability (*Frel*) of its metabolites (quantified as Quer) from the extract, relative to that from the pure rutin (Equations (1)–(3)).

(1) $$Fmet=\frac{{AUC}_{0–\infty \;metabolites}}{{AUC}_{0–\infty \;rutin}},$$

(2) $$F=\frac{{AUC}_{0–\infty \;p.o.}}{{AUC}_{0–\infty \;i.v.}}\times \frac{{Dose}_{i.v.}}{{Dose}_{p.o.}},$$

(3) $$Frel=\frac{{AUC}_{0–\infty \;QUER-EXT}}{{AUC}_{0–\infty \;QUER-RUT}}\times \frac{{Dose}_{RUT}}{{Dose}_{EXT}}.$$

In these equations, *AUC*_0–∞ *metabolites*_ is the area under the curve of Quer (representing rutin metabolites) expressed as equivalent to rutin. *AUC*_0–∞ *p.o.*_ and *AUC*_0–∞ *i.v.*_ are the area under the curve of rutin after oral or intravenous administration of a dose of the flavonoid, respectively. *AUC*_0–∞ *QUER-EXT*_ and *AUC*_0–∞ *QUER-RUT*_ are the area under the curve of Quer after the administration of the extract or pure rutin, respectively. All the AUC values were extrapolated to infinity. *Dose_i.v._* and *Dose_p.o._* are the doses of rutin intravenously or orally administered, respectively. *Dose_RUT_* and *Dose_EXT_* are the administered doses of pure rutin or the equivalent to rutin in the dose of the extract, respectively.

To preliminary evaluate possible matrix effects of the extract on the PK of rutin and its metabolites in the rabbits, parameters from NCA analysis were compared between pure rutin and extract using Student’s *t*-test or the Wilcoxon test when data did not fit a normal distribution. These tests were conducted using Statgraphics Centurion XVI v.16.1.02 software (Statpoint Technologies, The Plains, VA, USA). Differences were considered significant for *p*-values < 0.05.

#### 2.3.2. PopPK Analysis

PopPK analysis was performed using the Monolix^®^ module of the MonolixSuite™. Two models were established, one for rutin after i.v. administration of the treatments, and one for Quer, produced by deconjugation of Q3OG and Q3OS, after p.o. administration of the treatments.

Structural models with one or two compartments and first-order elimination were assessed, incorporating a double first-order absorption for p.o. experiments. An analysis of covariate was conducted, using the source of rutin as categorical covariate (pure compound or extract), which was incorporated into the model if statistically significant (*p* < 0.05 for Pearson’s and Wald tests with stochastic approximation). Correlations between random effects of the parameters were also examined and included in the model when significant (*p* < 0.05 for *t*-test). The final model, along with the residual error model, was chosen based on criteria including individual fit plots, Akaike’s information criteria (AIC) values, observation-versus-prediction plot analysis, precision of estimated parameters, and evaluation of residual distribution. 

The variability on fixed-effect model parameters was described by Equation (4):

(4) $$P_{i}=P_{pop}+\beta +\eta_{\left(i,p\right)},$$

where *P_i_* is the value of the individual PK parameter, *P_pop_* is the value of the population PK parameter, *β* represents the variability due to the covariate extract if significant, and *η* is the individual deviation with respect to the population. Models were parametrized in micro-constants, i.e., elimination rate constant (*k*), distribution rate constant from compartment 1 to compartment 2 (*k*_12_), and distribution rate constant from compartment 2 to compartment 1 (*k*_21_). When necessary, *Cl* was calculated as follows:

(5) Cl=k×V,

where *V* is the volume of distribution of first compartment. 

The model was internally validated through the visual predictive check (VPC) with 1000 simulated data points. Validation of structural, variability, and covariate models was considered achieved if the observed percentiles remained within the corresponding prediction intervals estimated across all simulated data and computed with a level of 90%. In addition, the parameter uncertainty was assayed by bootstrap analysis using 1000 runs. The model was considered stable, robust, and accurate if the mean parameters of the model were close to those estimated via bootstrap and fell within the 95% confidence interval (CI 95%) [25]. 

#### 2.3.3. Allometric Scaling

Individual pharmacokinetics parameters estimated by PopPK analysis in this study, along with those from a previous study conducted by the group in Wistar rats [17], were used to extrapolate *V* and *Cl* to humans using the simple allometry Equation (6):

(6) $$Y=a\times W^{b},$$

where *Y* is the PK parameter in humans, *W* is the human weight, typically set at 70 kg, and *a* and *b* are the coefficient and the exponent of scaling, respectively. These last two values are obtained from the relationship between the PK parameter and body weight across different species. Theoretically, *b* is fixed to 0.75 for *Cl* and 1.0 for Vd, although it can vary depending on the drug and population under study [26]. 

## 3. Results

### 3.1. Plasma Concentration–Time Profile of Rutin

**Figure 2:**
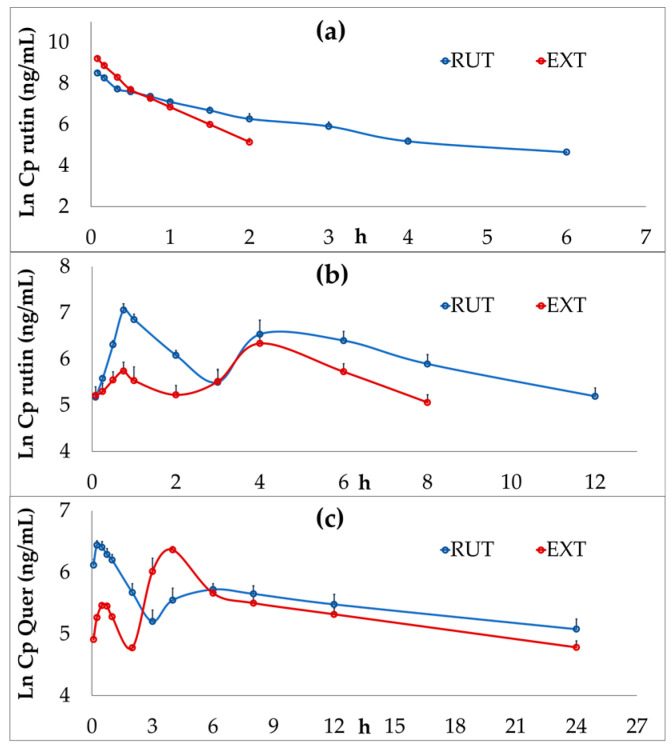
Semi-logarithmic plot of mean plasma concentration–time profiles. (**a**) Plasma concentrations of rutin following intravenous administration of pure rutin (RUT, 0.37 mg/kg) or *P. peruviana* extract (EXT, equivalent to 1.48 mg/kg of rutin). (**b**) Plasma concentrations of rutin after oral administration of RUT (100 mg/kg) or EXT (equivalent to 7.4 mg/kg of rutin). (**c**) Plasma concentrations of quercetin (Quer, representing rutin conjugated metabolites) after oral administration of RUT or EXT at the same doses as in (**b**).

After the i.v. administration, rutin levels in the animals were quantified within a concentration range from 4960 ± 340 to 105 ± 24 ng/mL (mean ± SEM) up to 6 h post-dosage for pure compound, and from 9940 ± 685 to 172 ± 22 ng/mL up to 2 h post-dosage for the extract, which was administered at doses equivalent to four times greater than the pure compound. In both cases, the semi-log plot is characterized by a decline in concentration that appears to be biphasic (Figure 2a). Meanwhile, free Quer was not detected in any of the original samples.

In p.o. experiments, plasma levels of rutin exhibited double peaks of concentrations at 0.75 and 4.0 h (Figure 2b), with *Cmax* occurring at the first peak for the pure compound (dose 100 mg/kg) and at the delayed peak for the extract (dose equivalent to 7.4 mg/kg of rutin).

Similar to the i.v. experiments, free Quer was not detected in the plasma after p.o. administration of either treatment. However, it was observed after the deconjugation reaction, indicating the presence of Q3OG and Q3OS from the first sampling time up to 24 h post-dosage. In this case, double peaks were also present, with the *Tmax* of Quer from the extract occurring at the second peak (Figure 2c).

### 3.2. Noncompartmental Analysis

According to NCA, the PK of pure rutin in the rabbits was characterized by a *Vss* of 0.290 ± 0.053 L/kg, a *Vz* of 0.084 ± 0.028, a *Cl* of 0.079 ± 0.013 L/h/kg, and an *F* as low as 0.006. Statistically significant changes were observed in these parameters when the extract was administered, including a 3.1-fold increase in *Vss*, a 2.1-fold increase in *Vz*, a 3.8-fold increase in *Cl*, and a substantial 28.3-fold increase in *F*, although it remains below 20% (Table 1).

Consistent with the increase in *F*, dose-normalized values of *AUC*_0–∞_ and *Cmax* of rutin showed significant increases when the extract was orally administered, with values rising by at least 6.8 times compared to the compound. However, the flavonoid exhibited more extensive metabolism with the extract, as indicated by a 1.6-fold increase in the *Fmet* (Table 1).

For both the pure compound and the extract administered orally, the systemic exposure of the metabolites, Q3OG and Q3OS, quantified as Quer, was greater than that of the parent compound, rutin. Consequently, the subsequent data analysis from the oral experiments focused on Quer.

The dose-normalized *AUC*_0–∞_ and *Cmax* of Quer after the p.o. administration of the extract also exhibited a significant increase compared to pure rutin. This enhancement is associated with a 12-fold rise in the bioavailability of the metabolites of the marker compound. Additionally, there were decreases in *Vz/F* and *Cl/F* from the compound to the extract and a notable shift in *Tmax*, with the second peak delayed by more than 3 h (Table 2).

### 3.3. PopPK Analysis

**Figure 3:**
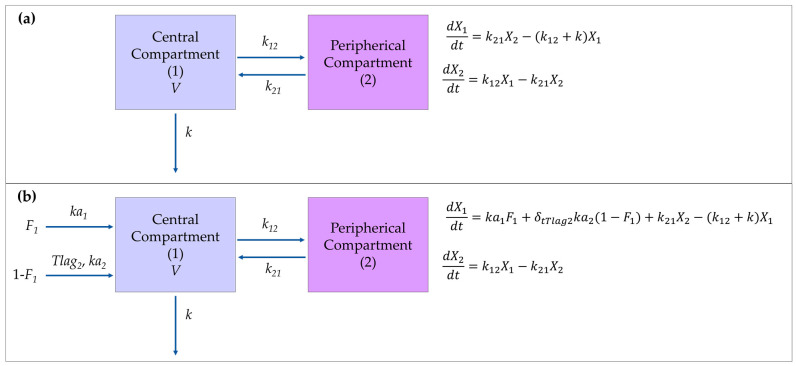
Schematic representation of the structural population models, parameters, and differential equations. (**a**) Two-compartment first-order elimination model for intravenous rutin. (**b**) Double first-order absorption model with two distribution compartments and first-order elimination for quercetin, representing the metabolites of rutin, quercetin*-3-O-*glucuronide, and quercetin*-3-O-*sulfate, after oral administration of rutin. *X*_1_: amount of the drug in the central compartment. *X*_2_: amount of the drug in the peripherical compartment. *V*: volume of distribution of first compartment. *k*: first-order elimination rate constant. *k*_12_: distribution rate constant from compartment 1 to compartment 2. *k*_21_: distribution rate constant from compartment 2 to compartment 1. *F*_1_: quercetin fraction absorbed at the first absorption site. ${ka}_{1}$: first-order absorption rate constants at first absorption site. ${ka}_{2}$: first-order absorption rate constants at second absorption site. *δ_tTlag_*_2_: function taking the value 0 if t < *Tlag*_2_ or 1 if t > *Tlag*_2_. *Tlag*_2_: delay time for the second absorption.

After the i.v. administration of either the pure compound or the extract, the plasma concentration–time profiles of rutin in rabbits best fit to a two-compartment model with first-order elimination. Figure 3a schematizes the parameters describing the model and the associated differential equations. The covariate analysis indicated that the matrix of the extract introduces significant variability in the subject population for *V*, *k*, and *k*_12_. No correlations among these parameters were detected. Consequently, a value of *β* was estimated for each one, representing the magnitude of changes attributable to the extract (Table 3).

**Figure 4:**
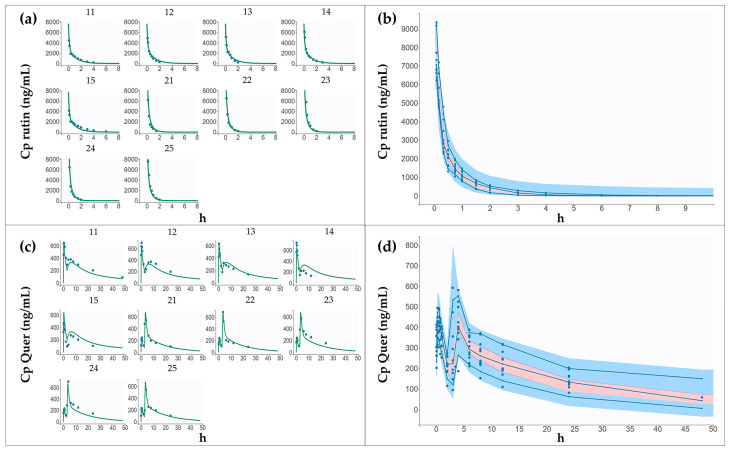
Individual data fit and visual predictive check (VPC) of the population models for rutin alone and the extract of calyces from *Physalis peruviana*. (**a**) Individual data fit to the population model for intravenous rutin (alone and in the extract). Dots represent data and lines are the predicted profile given by the estimated population model (**b**) VPC of model for intravenous rutin. Blue dots are the data, and blue lines are the empirical 10th, 50th, and 90th percentiles. Blue and red shaded areas are the prediction intervals for each percentile, based on 1000 simulated datasets. (**c**) Individual data fit to population model for quercetin, representing rutin metabolites after oral administration of rutin (alone and in the extract). (**d**) VPC of model for quercetin. The red circle indicates an outlier.

The final model used a proportional error approach with lognormal transformation of the data, except for *V*, where a logit-normal transformation was more appropriated. Under this model configuration, the AIC value achieved was 1159. A strong fit of the data to the model was confirmed by the population-fitted plasma profiles showed in Figure 4a, and by the individual observation-versus-prediction analysis, which indicated an outlier proportion of less than 6%. Additionally, the relative standard errors (RSEs) of the population parameters were all below 20.0% (Table 3), the residuals showed a random distribution around zero, and the VPC graphic did not exhibit any outlier zones (Figure 4b). Validation of the model was further confirmed by the IC 95% from the bootstrap analysis, which included the mean population values of the parameters, with a bias between means of less than 17% (Table 3).

The final equations for the parameters were as follows:

(7) $$log\;(\frac{V}{1-V})=log\;(\frac{V_{pop}}{1-V_{pop}})+\beta_{V}$$

(8) $$log\;(k)={log\;(k}_{pop})+\beta_{k}+\eta_{k}$$

(9) $$log\;(k_{12})={log\;(k}_{12pop})+\beta_{k_{12}}+\eta_{k_{12}}$$

(10) $$log\;(k_{21})={log\;(k}_{21pop})+\eta_{k_{21}}$$

The population parameters of rutin along with the *β* values, are presented in Table 3. Both *β_V_* and *β_k_* showed positive values, indicating an increase in these population parameters due to the extract. An opposite effect was observed on *k*_12_, as evidenced by a negative $\beta_{k_{12}}$ value.

A model consisting of double first-order absorption, two-compartmental distribution, and first-order elimination was established for Quer, derivate from Q3OG and Q3OS, in the oral experiments. The parameters and the differential equations of this model are illustrated in Figure 3b. The model includes the parameters *F*_1_, which represents the fraction of Quer (i.e., rutin metabolites) absorbed at the first absorption site, and *Tlag*_2_, denoting the delay of the second absorption. In this scenario, the extract significantly influenced the parameters related to the absorption and distribution of the compounds. Specifically, the extract affected the two absorption rate constants (${ka}_{1}$ and ${ka}_{2}$), the *V*, and the two micro-constants of distribution (*k*_12_, *k*_21_). Again, no correlations among the changing parameters were detected, and *β* values were estimated for each one (Table 4).

For this oral model, the residual error was better described by a combined model, resulting in an AIC value of 1244. The individual plasma profiles fit well to the population predictions (Figure 4c), with a proportion of outliers less than 9% in the observation-versus-prediction analysis. Residuals exhibited a random distribution, and only a few outlier dots were observed in the VPC graphic (Figure 4d). Additionally, there was consistency between population parameters and bootstrap estimates (Table 4). All these observations indicate the model validation. 

The population parameters of Quer are presented in Table 4, all with RSE values below 33.0%. The final equations for the parameters were as follows, with a logit-normal transformation applied to *F*_1_ and a lognormal transformation for the other parameters:

(11) $$log\;({ka}_{1})={log\;(ka}_{1pop})+\beta_{{ka}_{1}}$$

(12) $$log\;({ka}_{2})={log\;(ka}_{2pop})+\beta_{{ka}_{2}}$$

(13) $$log\;(\frac{F_{1}}{1-F_{1}})=log\;(\frac{F_{1pop}}{1-F_{1pop}})+\eta_{F_{1}}$$

(14) $$log\;({Tlag}_{2})={log\;(Tlag}_{2pop})+\eta_{{Tlag}_{2}}$$

(15) $$log\;(V)={log\;(V}_{pop})+\beta_{V}$$

(16) $$log\;(k)={log\;(k}_{pop})+\eta_{k}$$

(17) $$log\;(k_{12})={log\;(k}_{12pop})+\beta_{k_{12}}$$

(18) $$log\;(k_{21})={log\;(k}_{21pop})+\beta_{k_{21}}$$

### 3.4. Allometric Scaling

The predicted human *V* and *Cl* of rutin from both the pure compound and the extract are shown in Table 5. According to the allometric exponent obtained, there was an isometric relationship between rats and rabbits for *V* from the extract and for *Cl* from the pure compound. Meanwhile, *V* of the pure compound increased more slowly than body weight (negative allometry), and the *Cl* of the extract increased out of proportion to body weight (positive allometry). Parameters for humans were estimated using both calculated and theoretical values for exponent *b* (Equation (6)). The increase in the parameters for the flavonoid in the extract compared to the pure compound ranged from 2- to 3-fold for *V* and 3- to 9-fold for *Cl*.

## 4. Discussion

In a previous study, a preclinical PK investigation of a new ethanolic extract of calyces from *P. peruviana* with hypoglycemic activity was conducted in rats [17]. Since the preclinical stage of pharmaceuticals requires supporting data from at least two animal species, including rodents and nonrodents [6], this research conducted a similar study in New Zealand rabbits. This animal model presents convenient characteristics for PK studies, such as its manageable size, a gastrointestinal pH range similar to humans (from 1 to 7.4), and intestinal motility regulated by the motilin hormone, as in humans [27]. Several other studies have used this model to study the PK of plant extracts [28], plant isolated bioactive compounds [29], and, particularly, to study PK herb–drug interactions [30,31].

Changes in the PK of rutin between extract and pure compound were noticeable in the rabbits, even by simply observing the plasma concentration–time profiles after i.v. administration, as rutin from the extract exhibited lower plasma concentrations relative to the administrated dose and less time above detectable levels. These observations were confirmed by NCA, showing an increasing in Vd terms (*Vss* and *Vz*) and *Cl*. Similarly, the popPK model showed the differences in the PK of rutin due to the extract, described through the parameters *β_V_* and *β_k_*. The same behavior was observed in our previous study in rats [17], where it was hypothesized that the mixture of different compounds in the extract could saturate plasma proteins, increasing the free fraction of rutin that would be available for wider distribution in tissues and elimination. This hypothesis is supported by the PK literature [32,33].

Unlike in rats, plasma levels of rutin were detected in rabbits following oral administration of the treatment, although the bioavailability remained very low, even from the extract, which produced a higher *F*. This low bioavailability of rutin was an expected result, considering its characteristics as a very slightly soluble substance with high efflux from enterocytes and extensive metabolism in the gut [34,35,36]. There, it becomes its aglycone, Quer, which is further metabolized in enterocytes, mainly producing Q3OG and Q3OS [36,37]. In fact, rutin has been classified as Class IV in the Biopharmaceutical Classification System (low solubility and low permeability) [23]. 

The greater *Fmet* of rutin along with the greater *Frel* of Quer (representing rutin metabolites Q3OG and Q3OS) from the extract is also consistent with previous results from our group in comparative permeability studies using the Caco-2 model. In those studies, a greater presence of Q3OG and Q3OS was observed on the basolateral side in the experiments with the extract compared to the pure compound. This was associated with enhanced uptake of rutin in enterocytes, resulting from reduced drug efflux, likely due to P-glycoprotein inhibition mediated by other compounds in the extract [23].

In the popPK analysis, Quer was well fitted to a double absorption model (Figure 3), which is supported by the complex mechanism of enteric circulation described for flavonoids with conjugated metabolites. According to this mechanism, these metabolites can be directly effluxed into the lumen and subsequently hydrolyzed to their aglycones by intestinal microflora enzymes, whose activity is higher in the large intestine, the site from which aglycones are reabsorbed [38]. In this case, it is assumed that a fraction of rutin is absorbed in the small intestine as Q3OG and Q3OS (*F*_1_ in the model). However, the majority of the conjugates are effluxed and then hydrolyzed to Quer in the large intestine of the rabbits, where it partially enters the enterocytes, is further conjugated, and the remaining bioavailable conjugates (1-*F* in the model) are transported to the blood. Thus, the two absorption processes of rutin metabolites occur at different sites, rates (${ka}_{1}$ and ${ka}_{2}$ in the model), and times (*Tlag*_2_ in the model), resulting in the double peak observed in the concentration–time profiles. The parameterization of the double-peak phenomenon was clearly an advantage of the popPK model compared to NCA, which is unable to account for it. 

Based on this model, the extract increases the second absorption phase of rutin metabolites, explaining their greater *Frel*. This change due to the extract was described in the popPK model through the parameter $\beta_{{ka}_{2}}$. Such changes could result from reduced drug efflux, as mentioned earlier [23], but might also indicate that the matrix of the extract preferentially facilitates the enteric recirculation of rutin metabolites.

As is known, plant extracts contain a complex mixture of compounds. Some authors have identified Quer and glycosides of Quer rather than rutin in alcoholic extracts of calyces from *P. peruviana* [39], which could account for the increase observed in the *Frel* of Q3OG and Q3OS. However, for the extract used in this research, fractionation followed by NMR and MS spectroscopy analysis did not reveal the presence of these other compounds [19,22]. Even if these compounds were present, their presence alone would not be sufficient to explain the changes observed in the plasma concentration–time profiles. 

Firstly, changes in primary PK parameters of rutin, along with an increase in its bioavailability, were confirmed from data obtained by directly measuring this analyte, whose dose in the extract was well known because it was quantified by a validated analytical method [20]. Secondly, all plasma samples were analyzed before deconjugation reactions, and no Quer was detected in any of them. Thirdly, rutin is clearly the main compound in the extract, as shown in the chromatographic profile available in the supplementary information of the previous publication [17]. Therefore, the changes in PK parameters observed could be considered as an effect of the matrix of the extract. 

The reduction in *Vz*/*F* and *Cl*/*F* of Quer due to the extract, observed in the NCA, could be interpreted as a result of the increased systemic exposure of rutin metabolites rather than an actual reduction in their primary Vd or *Cl*. However, the popPK model allowed for the estimation of primary *V* for Quer, i.e., independent of *F*, and identified a decrease in this parameter due to the extract. Meanwhile, no significant changes were observed in *k* in either the NCA or popPK, meaning that the reduction in *Cl* is likely due to changes in *V*.

In this study, the PK parameters of rutin in rabbits were reported, to the best of our knowledge, for the first time. This information could be valuable for researchers studying the pharmacokinetics of botanical drugs. This study was conducted to help design a new formulation and dosage for the ethanolic extract of calyces from *P. peruviana*, in order to evaluate its clinical effect in human in future studies. The generated knowledge led to obtaining a first approximation of human *V* and *Cl* for pure rutin and in the extract by simple allometry. The exponent *b* resulting from the scaling of *V* is reasonably close to the theoretical value, especially for the extract. However, for *Cl*, it may be necessary to include a third animal species or consider other aspects of scaling, such as maximal lifespan potential or brain weight, to improve the prediction from animals to humans for this parameter [40]. Currently, our group is focusing on simulating the pharmacokinetics of rutin and the extract in humans, using a physiologically based pharmacokinetics approach (PBPK) to further advance the development of this new medicinal product. 

## 5. Conclusions

In general, rutin exhibits low *V*, high *Cl*, and low *F* in rabbits, with its metabolites Q3OG and Q3OS being more bioavailable. The matrix of the extract results in increased values for all three parameters, as well as enhanced the systemic exposure of rutin metabolites. The PK interactions of the extract matrix were detected by NCA and popPK modeling. The model was able to explain the double peak phenomenon of rutin metabolites and define the equations describing the change in the PK parameters due to the extract. The results of this research will be useful in continuing the development process of a new herbal medicine. 

## Figures and Tables

**Table 1:** Pharmacokinetics parameters of rutin alone and in the extract of calyces from *P. peruviana* in rabbits by noncompartmental analysis.

Parameter	Intravenous Administration (i.v.)		Oral Administration (p.o.)	
	Pure Rutin (0.37 mg/kg)	Extract (Equivalent to 1.48 mg/kg of Rutin)	Pure Rutin (100 mg/kg)	Extract (Equivalent to 7.4 mg/kg of Rutin)
*AUC*_0–∞_ (ng·h/mL)	4740.94 ± 813.800	5108.77 ± 1119.320	7792.96 ± 2109.620	4483.97 ± 755.300
*Cp*^0^ or *Cmax* (ng/mL)	4960.02 ± 760.234	9939.91 ± 1531.172	1166.2 ± 370.570	599.43 ± 197.640
*Tmax* (h)	-	-	0.75 ± 0.000	3.35 ± 1.450
*Vss* (L/kg)	0.29 ± 0.053	0.91 ± 0.320 *	-	-
*Vz* or *Vz/F* (L/kg)	0.084 ± 0.028	0.18 ± 0.055 *	59.08 ± 11.416	5.20 ± 1.470 *
*Cl* or *Cl/F* (L/h/kg)	0.079 ± 0.013	0.300 ± 0.060 *	13.59 ± 3.564	1.69 ± 0.267 *
*λz* (h^−1^)	1.06 ± 0.464	1.79 ± 0.302	0.234 ± 0.066	0.33 ± 0.047 *
*t*_1/2_ (h)	0.79 ± 0.381	0.40 ± 0.070	-	-
*MRT* (h)	3.63 ± 0.163	2.94 ± 0.515 *	11.76 ± 0.916	14.89 ± 1.690 *
*AUC*_0–∞_*/dose* (h/L/kg)	12.90 ± 2.214	0.035 ± 0.008 *	0.078 ± 0.021	0.61 ± 0.102 *
*Cp*^0^ or *Cmax/dose* (L^−1^/kg)	13.49 ± 2.068	0.067 ± 0.010 *	0.012 ± 0.004	0.081 ± 0.026 *
*Fmet*	-	-	2.38 ± 0.411	3.80 ± 0.749 *
*F*			0.006	0.17

Data are expressed as the mean ± standard deviation of *n* = 5. *AUC*_0–∞_: area under the curve extrapolated to infinite. *Cp*^0^: plasma concentration at zero time for i.v. *Cmax*: peak concentration for p.o. *Tmax*: time for peak concentration. *Vss*: volume of distribution at the steady state. *Vz*: volume of distribution at the terminal phase for i.v. *Vz/F*: volume of distribution at the terminal phase divided by bioavailability for p.o. *Cl*: clearance for i.v. *Cl/F*: clearance divided by bioavailability for p.o. *λz*: first-order rate constant associated with the terminal portion of the curve. *t*_1/2_: terminal half-life. *MRT*: mean residence time. *Fmet*: metabolized fraction, calculated as the ratio between *AUC*_0–∞_ values for rutin metabolites and rutin. *F*: Absolute bioavailability. * Significantly different from pure rutin at the same administration route (*p* < 0.05).

**Table 2:** Pharmacokinetics parameters of quercetin ^1^ after oral administration of pure rutin or an extract of calyces from *P. peruviana* in rabbits by noncompartmental analysis.

Parameter	Pure Rutin (100 mg/kg)	Extract (Equivalent to 7.4 mg/kg of Rutin)
*AUC*_0–∞_ (ng ·h/mL)	9278.77 ± 3275.282	8273.24 ± 1065.429
*Cmax* (ng/mL)	640.88 ± 102.922	613.95 ± 81.396
*Tmax* (h)	0.35 ± 0.137	3.90 ± 0.224 **
*Vz/F* (L/kg)	226.60 ± 53.826	21.44 ± 5.282 *
*Cl/F* (L/h/kg)	11.98 ± 4.405	0.91 ± 0.113 *
*λz* (h^−1^)	0.054 ± 0.022	0.043 ± 0.006
*MRT* (h)	21.70 ± 4.513	22.12 ± 1.915
*AUC*_0–∞_*/dose* (h/L/kg)	0.093 ± 0.033	1.12 ± 0.144 *
*Cmax/dose* (L^−1^/kg)	0.0064 ± 0.001	0.083 ± 0.011 *
*Frel*	-	12.0

^1^ Quercetin, representing the metabolites of rutin quercetin*-3-O-*glucuronide and quercetin*-3-O-*sulfate, is obtained through deconjugation reaction of them. *AUC*_0–∞_: area under the curve extrapolated to infinite. *Cmax*: peak concentration. *Tmax*: time for peak concentration. *Vz/F*: volume of distribution at the terminal phase divided by bioavailability. *Cl/F*: clearance divided by bioavailability. *λz*: first-order rate constant associated with the terminal portion of the curve. *MRT*: mean residence time. *Frel*: relative bioavailability, calculated as the ratio between *AUC*_0–∞_ values for rutin metabolites from the extract and from the pure compound. Data are expressed as the mean ± standard deviation of *n* = 5. * Significantly different from pure rutin (*p* < 0.05). ** Significantly different from pure rutin (*p* < 0.01).

**Table 3:** Population pharmacokinetics parameters of rutin alone and in the extract of calyces from *P. peruviana* in rabbits.

Parameter	Model Estimations		Bootstrap Analysis (*n* = 1000 ^1^)		
	Population Value	R.S.E.	Mean	CI 95%	%Bias
*V* (L/kg)	0.048	5.2	0.046	0.040–0.054	−4.68
*β_V_*	0.678	9.6	0.710	0.50–0.89	4.61
*k* (h^−1^)	1.924	9.1	2.027	1.58–2.32	5.34
*β_k_*	0.625	19.7	0.585	0.34–0.85	−6.35
*k*_12_ (h^−1^)	3.666	19.3	4.095	2.62–5.34	11.71
$\beta_{k_{12}}$	−0.634	43.6	−0.737	−1.2–−0.23	16.21
*k*_21_ (h^−1^)	3.777	9.7	3.801	3.18–4.77	0.62
**Standard deviation of the Random Effects**					
*Ω k*	0.173	23.0	0.154	0.099–0.20	−11.06
*Ω k*_12_	0.362	26.4	0.326	0.14–0.45	−10.08
*Ω k*_21_	0.266	27.3	0.239	0.099–0.038	−10.29
**Error model parameter**					
*b*	0.076	9.8	0.072	0.061–0.083	−5.21

Data from intravenous administration of pure rutin (0.37 mg/kg) or extract (equivalent to 1.48 mg/kg of rutin). *V*: volume of distribution of first compartment. *k*: first-order elimination rate constant. *k*_12_: distribution rate constant from compartment 1 to compartment 2. *k*_21_: distribution rate constant from compartment 2 to compartment 1. *β*: variability attributable to the covariate extract. *Ω*: random variability. *b*: parameter for proportional error model. R.S.E.: relative standard error. %Bias: (mean − population value)/population value × 100. ^1^ Number of runs that did not converge: 177.

**Table 4:** Population pharmacokinetics parameters of quercetin representing rutin metabolites after oral administration of pure rutin or the extract of calyces from *P. peruviana* in rabbits.

Parameter	Model Estimations		Bootstrap Analysis (*n* = 1000 ^1^)		
	Population Value	R.S.E.	Mean	CI 95%	%Bias
${ka}_{1}$ (h^−1^)	11.146	6.3	11.179	9.92–12.34	0.26
$\beta_{{ka}_{1}}$	−0.949	18.1	−1.013	−1.81–−0.71	6.67
${ka}_{2}$ (h^−1^)	0.094	21.0	0.0930	0.071–0.12	−1.28
$\beta_{{ka}_{2}}$	3.528	6.7	3.628	2.78–4.56	2.84
*F*_1_	0.270	9.4	0.271	0.25–0.30	0.24
*Tlag*_2_ (h)	2.971	2.8	2.988	2.88–3.18	0.58
*V* (L/kg)	0.036	14.8	0.036	0.031–0.042	0.32
*β_V_*	−1.910	8.0	−1.960	−2.65–−1.72	2.64
*k* (h^−1^)	0.221	20.6	0.225	0.16–0.35	1.61
*k*_12_ (h^−1^)	0.251	28.3	0.261	0.19–0.39	4.02
$\beta_{k_{12}}$	1.076	33.5	1.117	0.46–2.27	3.77
*k*_21_ (h^−1^)	0.040	32.4	0.043	0.012–0.098	9.42
$\beta_{k_{21}}$	2.067	16.0	2.138	1.07–3.30	3.42
**Standard deviation of the Random Effects**					
*Ω F*_1_	0.160	23.9	0.143	0.04–0.24	−10.72
*Ω Tlag*_2_	0.077	33.3	0.064	0.0042–0.13	−16.40
*Ω k*	0.389	23.9	0.360	0.23–0.48	−7.54
**Error model parameter**					
*a*	20.661	15.4	20.070	8.44–25.6	−2.86
*b*	0.044	32.4	0.035	0–0.065	−20.14

Data from oral administration of pure rutin (100 mg/kg) or extract (equivalent to 7.40 mg/kg of rutin). ${ka}_{1}$: first-order absorption rate constants at first absorption site. ${ka}_{2}$: first-order absorption rate constants at second absorption site. *F*_1_: quercetin fraction absorbed at the first absorption site. *Tlag*_2_: delay time for the second absorption. *V*: volume of distribution of first compartment. *k*: first-order elimination rate constant. *k*_12_: distribution rate constant from compartment 1 to compartment 2. *k*_21_: distribution rate constant from compartment 2 to compartment 1. *β*: variability attributable to the covariate extract. *Ω*: random variability. *a* and *b*: parameters for combined error model. R.S.E.: relative standard error. %Bias: (mean − population value)/population value × 100. ^1^ Number of runs that did not converge: 100.

**Table 5:** Human pharmacokinetics parameters of rutin alone and in the extract of calyces from *Physalis peruviana* predicted by simple allometry.

Parameter	Rat Parameter	Rabbit Parameter	a Coefficient	b Exponent	Human Parameter (Experimental b) ^1^	Human Parameter (Theoretical b) ^2^
*V_RUT_* (L)	0.024	0.096	0.057	0.8	1.410	3.988
*V_EXT_* (L)	0.035	0.190	0.102	0.9	4.592	7.128
*Cl_RUT_* (L/h)	0.031	0.188	0.096	0.9	5.389	2.323
*Cl_EXT_* (L/h)	0.072	0.690	0.296	1.2	48.938	7.160

*V*: volume of distribution of first compartment. *Cl*: clearance. *RUT*: parameter for pure rutin. *EXT*: parameter for rutin in the extract. ^1^ Parameter calculated using b exponent from the data. ^2^ Parameter calculated using theoretical *b* exponent, 1.0 for *V* and 0.75 for *Cl*.

## Data Availability

Publicly available datasets were analyzed in this study. These data can be found here: https://repositorio.unal.edu.co/handle/unal/78924 (accessed on 1 March 2021). More information is available to interested researchers upon request from the corresponding author.

## Supplementary Materials

The following supporting information can be downloaded at: https://www.mdpi.com/article/10.3390/pharmaceutics16101241/s1, Figure S1: Selectivity of the bioanalytical method. Colored lines are blank samples from different rabbits; Table S1: Accuracy and precision of the method used for quantify rutin and quercetin in rabbit plasma.
