# Supplementary material for: Rutin and Physalis peruviana Extract: Population Pharmacokinetics in New Zealand Rabbits
Source: Pharmaceutics. 2024 Sep 24;16(10):1241. doi: 10.3390/pharmaceutics16101241 (PMC11510156; doi:10.3390/pharmaceutics16101241)
Supplement: Supplementary file 1 [file pharmaceutics-16-01241-s001.zip › pharmaceutics-3172795-Supplementary/pharmaceutics-3172795-supplementary.pdf]

Supplementary Materials: Rutin and *Physalis peruviana* Extract: Population Pharmacokinetics in New Zealand Rabbits

Gina Paola Domínguez Moré [1,2], Diana P. Rey [2], Ivonne H. Valderrama [2], Luis F. Ospina [2] and Diana Marcela Aragón [2,*]

[1] Centro de Servicios Farmacéuticos y Monitoreo de Fármacos, Facultad de Química y Farmacia, Universidad del Atlántico, Carrera 30 # 8–49, Puerto Colombia 081001, Colombia; ginadominguez@mail.uniatlantico.edu.co
[2] Departamento de Farmacia, Universidad Nacional de Colombia, Av. Carrera 30 # 45–03 Edif. 450, Bogotá 111321, Colombia; dpreyp@unal.edu.co (D.P.R.); ihvalderramap@unal.edu.co (I.H.V.); lfospinag@unal.edu.co (L.F.O.)
* Correspondence: dmaragonn@unal.edu.co; Tel.: +57-6013165000 (ext. 14630) or +57-3177488247

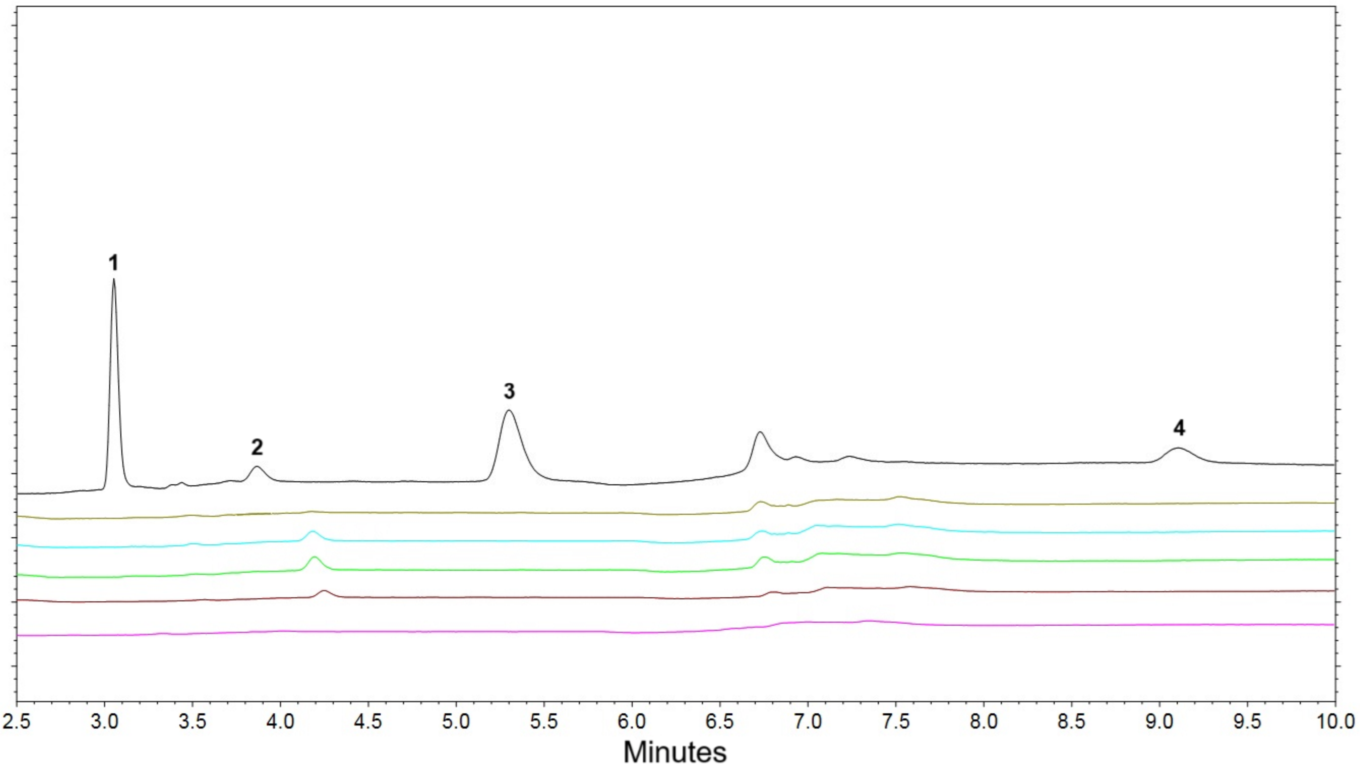

Figure S1. Selectivity of the bioanalytical method. Colored lines are blank samples from different rabbits. Black line is blank plasma spiked with rutin (250 ng/mL, peak 1), quercetin-3-O-glucuronide (50 ng/mL, peak 2), quercetin (250 ng/mL, peak 3), and the internal standard chrysin (1000 ng/mL, peak 4). It is noticed that there was not interfering signals for the analytes and IS. Column Kinetex® EVO C18, 2,6 µm, 100 × 2.1 mm at 30ºC, mobile phase with formic acid 0.1% (A) and acetonitrile acidified with formic acid at 0.5%, in gradient from 85 to 65% A, at 0.5 mL/min. Detection at 260 nm and injections of 6 µL of the samples. Samples were prepared by protein precipitation with methanol (1:2).

Table S1. Accuracy and precision of the method used for quantify rutin and quercetin in rabbit plasma

	Rutin				Quercetin			
**Quality control**	**LLOC**	**LQC**	**MQC**	**HQC**	**LLOC**	**LQC**	**MQC**	**HQC**
**Nominal concentration (ng/mL)**	100	250	5000	10000	100	250	5000	10000
**Between -run**								
**Calculated concentration (ng/mL)[1]**	90.1	252.8	5363.2	9608.1	99.7	247.8	5190.3	9806.6
**Recovery (%)[2]**	90.1	101.1	107.3	96.1	99.7	99.1	103.8	98.1
**CV (%)[3]**	9.7	3.0	8.6	2.7	14.8	2.0	5.4	4.7
**Within-run**								
**Calculated concentration (ng/mL)[1]**	119.2	268.7	5674.2	10233.4	102.2	251.7	5290.3	10307.9
**Recovery (%)[2]**	119.2	107.5	113.5	102.3	102.2	100.7	105.8	103.1
**CV (%)[3]**	3.6	7.4	3.9	4.0	8.8	9.8	10.3	6.2

LLOC: low limit of quantification. LQC: low quality control. MQC: medium quality control. HQC: high quality control. [1] Concentration calculated using the calibration curves $y = 0.00042x + 0.00910$ (r^2 0.9996) for rutin and $y = 0.00056x - 0.00767$ (r^2 0.99980) for quercetin. [2] Accuracy measured as percentage of calculated concentration relative to nominal concentration. [3] Precision measured as coefficient of variation. n = 5.
